# Road screening and distribution route multi-objective robust optimization for hazardous materials based on neural network and genetic algorithm

**DOI:** 10.1371/journal.pone.0198931

**Published:** 2018-06-21

**Authors:** Changxi Ma, Wei Hao, Fuquan Pan, Wang Xiang

**Affiliations:** 1 School of Traffic and Transportation, Lanzhou Jiaotong University, Lanzhou, China; 2 Hunan Key Laboratory of Smart Roadway and Cooperative Vehicle-Infrastructure Systems(No.2017TP1016), Changsha University of Science and Technology, Changsha, Hunan, China; 3 Key Laboratory of Safety Design and Reliability Technology for Engineering Vehicle, Changsha University of Science and Technology, Changsha, Hunan, China; 4 Hunan Provincial Key Laboratory of Intelligent Processing of Big Data on Transportation, Changsha University of Science and Technology, Changsha, Hunan, China; 5 School of Automobile and Transportation, Qingdao University of Technology, Qingdao, Shandong, China; Chongqing University, CHINA

## Abstract

Route optimization of hazardous materials transportation is one of the basic steps in ensuring the safety of hazardous materials transportation. The optimization scheme may be a security risk if road screening is not completed before the distribution route is optimized. For road screening issues of hazardous materials transportation, a road screening algorithm of hazardous materials transportation is built based on genetic algorithm and Levenberg–Marquardt neural network (GA-LM-NN) by analyzing 15 attributes data of each road network section. A multi-objective robust optimization model with adjustable robustness is constructed for the hazardous materials transportation problem of single distribution center to minimize transportation risk and time. A multi-objective genetic algorithm is designed to solve the problem according to the characteristics of the model. The algorithm uses an improved strategy to complete the selection operation, applies partial matching cross shift and single ortho swap methods to complete the crossover and mutation operation, and employs an exclusive method to construct Pareto optimal solutions. Studies show that the sets of hazardous materials transportation road can be found quickly through the proposed road screening algorithm based on GA-LM-NN, whereas the distribution route Pareto solutions with different levels of robustness can be found rapidly through the proposed multi-objective robust optimization model and algorithm.

## Introduction

Hazardous materials refer to products with flammable, poisonous, and corrosive properties that can cause casualties, damage to properties, and environmental pollution, and require special protection in the process of transportation, loading, unloading, and storage. In recent years, hazardous materials transportation accidents have occurred frequently, causing vehicle damages, fatalities, and environment pollution. Distribution route optimization of hazardous materials refers to the design of a safe and efficient distribution plan based on existing transportation network according to the characteristics of hazardous materials and transportation requirements. The result of this study can provide a direct reference for relevant decision-making departments. Preventing hazardous materials transportation accidents is crucial. Thus, research on hazardous materials distribution route optimization has great significance.

Many scholars have investigated hazardous materials transportation issues. Zografos and Davis proposed a multiple criteria shortest path problem and used an optimized target program to obtain the solution [1]. Karkazis and Boffey established a route optimization model of hazardous materials transportation with the aim of minimizing population risk and cost, and used branch and bound algorithm to perform numerical experiments [2]. Helander and Melachrinoudis combined the route problem with the expected number of hazardous materials transportation fatalities, and realized route optimization design of hazardous materials transportation [3]. Akgun et al. identified that studies on route choice problem of hazardous materials transportation had an important significance, and proposed a method to generate candidate route sets [4]. Kara and Verter designed a double goal and double deck programming model to obtain the least number of hazards and the shortest transportation route [5]. Zografos and Androutsopoulos defined the hazardous materials transportation problem as a double-objective route problem, the goal of which is to minimize the risk and cost, and proposed a new heuristic algorithm [6]. Meng et al. established a multi-objective route optimization model of hazardous materials with time constraints, and used dynamic programming method to solve the problem based on the case study [7]. Liu et al. built a fuzzy comprehensive evaluation model using multilevel fuzzy comprehensive evaluation method to optimize the transportation route [8]. Akgun et al. established a route optimization model to minimize the risk and cost, considered the effect of road attributes on the risk, and performed relative experiments [9]. Erhan Erkut and Osman ALP built a hazardous materials transportation model in which three factors are considered, namely, accident rate, population exposure number, and running time, and proposed quasi-polynomial dynamic programming algorithm to solve the model [10]. Dadkar et al. realized that the diversity of transport routes can provide the opportunity for the driver to switch routes to avoid the same population at risk, established route optimization model, and applied heuristic algorithm to solve the model [11]. Erkuta and Gzara proposed a model similar with [4] that completed the transport network decision of single origin destination point by minimizing transportation risk and transportation costs [12]. Shen studied the multi-objective route optimization problem of hazardous materials through analyzing actual accident cases [13]. Renee and Mary established a new transportation risk analysis model and Bayesian network decision model based on the existing hazardous materials transportation data, which have a certain auxiliary function for transportation network optimization [14]. Verma built a double-objective optimization model with the aim of minimizing the risk and cost, and implemented cost boundary algorithm to solve the problem [15]. Jassbi et al. investigated the multi-objective optimization framework of hazardous materials transportation, which includes the shortest mileage, the least number of residents, the minimum social risk, and the minimum accident probability [16]. Vasiliki Kazantzi et al. established a transportation route optimization model of hazardous materials with transportation risk and cost considered, and applied the Monte Carlo method in simulation. The results of the study have a specific reference value for the optimization design problem of transportation network with single origin destination point [17]. Xie and Travis Waller proposed advanced labeling algorithm to solve the double-objective transportation route optimization model with single origin destination point [18]. Das et al. examined the transportation route problem of transportation network with limited capacity, and found non-dominated solutions from the multi-objective algorithm for multiple starting and end points transportation network [19–20]. Ma et al. analyzed the transportation route choice problem under certain and uncertain environments, and proposed the multi-objective route planning model of certain environment, multi-objective route chance constrained model, and multi-objective route opportunities dependent model of uncertain environment [21–23]. Pradhananga et al. built a double-objective transportation route optimization model with time window where minimum transportation time and transportation risk are considered as the optimization objective, and designed a heuristic algorithm of searching Pareto optimal solution [24].

In view of the abovementioned studies, some interesting results are obtained in the transportation network optimization of hazardous materials. However, three problems are found as follows:

(1) The abovementioned research results have a certain degree of adaptability for transportation route optimization of single origin destination point, but do not have strong adaptability for multiple origin destination points.
(2) Not all road sections are suitable for hazardous materials transportation. Hence, road screening is necessary to remove road sections unsuitable for hazardous materials transportation before the distribution route optimization. Otherwise, the obtained optimization scheme may cause critical security risk. The road screening route choice algorithm should be built because the roads are not determined by road situation or decision-maker experience. The algorithm can provide a security based network for route optimization and low complexity of solving the model.
(3) The transportation risk value of each road section should be determined before distribution route optimization of hazardous materials. However, the value is uncertain because of the limitations of the statistical data method and time-variant characteristics of transportation risk. When the risk is assumed for a certain or a random number, which is considered as the initial condition. Models are then built by using traditional optimization methods or stochastic chance-constrained programming method. The effect of uncertain data on the quality and feasibility of the model is not considered in these methods. Thus, the obtained optimization scheme poses significant risk in practical applications.

Ben and Nemirovski indicated that small uncertain input data may incur considerable costs in the practical application of traditional optimal solution [25–26]. For hazardous materials transportation network, the losses are the money and fatalities. The robust optimization model and robust optimal solution are expected to solve this problem. The robust optimization method is a powerful tool for solving the uncertain optimization problem, which describes uncertainty by the set. Obtained robust solutions are feasible for any elements of the set, and have good adaptability to uncertainty. Therefore, analyzing the uncertainty of hazardous materials transportation risk, introducing the robust optimization theory to establish the robust optimization model, designing a protective solving method of uncertain data, and obtaining robust optimization solutions of distribution route by calculation are necessary. The adaptability of distribution plan for uncertainty can be improved to ensure that the optimized transportation network can effectively protect the safety of lives and property.

The distribution route optimization problem of hazardous materials is a typical multi-objective optimization problem. Designing suitable multi-objective algorithms is important. Niche Pareto genetic algorithm (GA) [27], non-dominated sorting GA [28], and strong Pareto evolutionary algorithm [29], are representative algorithms. These algorithms have improved solving efficiency for special problems. However, these algorithms cannot be applied directly for a specific problem. Hence, this paper designs a new multi-objective GA based on route optimization characteristics of hazardous materials transportation.

The rest of this paper is organized as follows: Section 2 studies the road screening algorithm of hazardous materials alternative route selection; Section 3 builds a transportation route multi-objective robust optimization model of hazardous materials; Section 4 designs a new multi-objective GA; Section 5 presents a case study; Section 6 provides the conclusion.

## Study of hazardous materials transportation road screening

### The necessity analysis of road screening and the screening method summary

The obtained optimization scheme may contain road sections unsuitable for hazardous materials transportation if transportation route optimization was performed before road screening. Road screening by using scientific methods and removing unsuitable road sections for hazardous materials transportation can guarantee the feasibility of transportation route optimization and vehicle scheduling optimization results, and reduce the difficulty of providing solutions to the problem (because of fewer road sections in the initial transport network). Therefore, studying the road screening problem of hazardous materials is necessary.

In this paper, a genetic Levenberg–Marquardt neural network(NN) that combines the advantages of genetic algorithm(GA) with Levenberg–Marquardt (LM) method, and is based on the analysis of various computing methods is built. First, the weights and threshold of the neural network are initialized by GA. Then, the neural network is trained and tested using the LM method. Finally, transportation network road screening is performed using the tested GA-LM-NN model to accelerate the convergence rate, improve prediction accuracy, and complete the road screening.

### Road screening index system of hazardous materials transportation

According to the transportation characteristics of hazardous materials and statistical data on traffic accidents, the index set of transportation route screening system is determined as *C* = {*c*_1,_*c*_2,_…,*c*_15_} decision objective D = {*d*_1,_*d*_2,_*d*_3_}, where *c*_1_ is the road width of unilateral motor vehicle; *c*_2_ is the number of small radius horizontal and vertical curves (Note: horizontal curve radius less than 100 m and vertical curve radius less than 500 m are called small radius); *c*_3_ is the minimum horizontal curve radius of road, the unit of which is m; *c*_4_ is the minimum vertical curve radius of road, the unit of which is m; *c*_5_ is the length of longitudinal slope more than 4%, the unit of which is m; *c*_6_ is the maximum longitudinal slope gradient; *c*_7_ is the width of central strip, the unit of which is m (Note: if there is a fence separating, *c*_7_ = 0.2 m; if no fence or green belt separating exists, *c*_7_ = 0; if a green belt separating exists, *c*_7_ is the actual width); *c*_8_ is the designed speed, the unit of which is km/h; *c*_9_ is the quality of pavement (the quality of pavement is divided in three kinds: good, medium, and general, where the corresponding values of *c*_9_ are 1, 2, and 3 successively; if the pavement is unqualified, it can be directly set to be excluded from the driving section); *c*_10_ is the clear degree of traffic signs and markings in the section (the degrees of traffic signs are divided in three: good, medium, and general, where the corresponding values of *c*_10_ are 1, 2, and 3 successively; if some sections do not have traffic signs and markings, it can be set to be excluded from the driving section); *c*_11_ is the number of limit value in the alignment index (highway and urban road design specifications require that five linear indexes, including the minimum half plane curve, the minimum length of plane curve, the minimum radius of vertical curve, vertical curve length, and the maximum longitudinal slope with limitation, can be lower than the general limit value. Thus, the values still conformed to the standards, but accident risks still exist); *c*_12_ is the number of intersection and entrance of the interference section; *c*_13_ is the width of the emergency parking area, the unit of which is m; *c*_14_ is the road traffic control environment (the road traffic control environment is divided in three: good, medium, and general, where the corresponding values of *c*_14_ are 1, 2, and 3 successively); *c*_15_ is the section average saturation; *d*_*i*_ refers to the status of road safety, which is divided into three levels: good safety, general safety, and bad safety, by using (1, 0, 0), (0, 1, 0), and (0, 0, 1) to measure (state of traffic safety can be determined according to the historical data of traffic accidents in each section)[30].

### A neural network model of hazardous materials screening system

**Fig 1:**
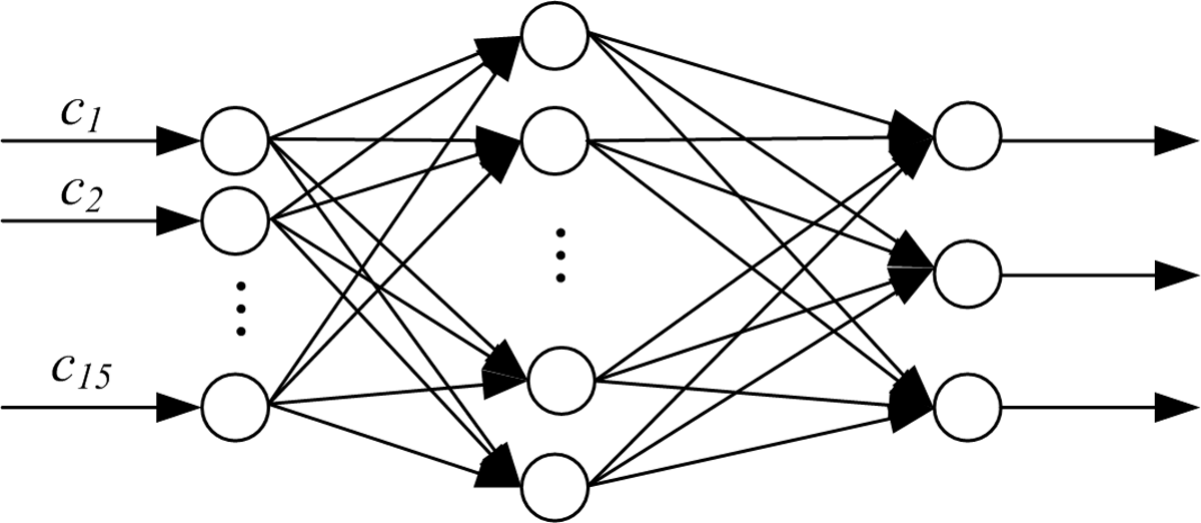
Road screening LM neural network model structure of hazardous materials transportation.

According to the above established prohibited section screening system, 15 input parameters and 3 output parameters are presented in the system. In this paper, we used a three-layer neural network and the specific structure of the neural network is 31–3–15 based on empirical formula. The neural network diagram is shown in Fig 1.

### Date dimensionless processing

The dimension of decision index in transportation road screening system is different. Three types of index, including benefit, cost, and interval, are used. Dimensionless processing of these data is needed before inputting the neural network.

Suppose the matrix composed of the collected data is *X* = (*x*_*ij*_)_m×n_, *i* = 1,2,…,*m*, *j* = 1,2,…,*n*, where *x*_*ij*_ is the actual value of attribute *j* in data set *i*, *m* is the number of sets, *n* is the number of condition attributes. Suppose $\underset{1\leq i\leq m}{max}x_{ij}=a_{j}$, where *a*_*j*_ is the maximum value of attribute *j*; $\underset{1\leq i\leq m}{max}x_{ij}=b_{j}$, *b*_*j*_ is the minimum value of attribute *j*. The situations of the benefit, cost, and interval types are stated as follow.

Situation 1: for the benefit type attribute index (the larger the index value, the better), the conversion formula is as follows:

$$y_{ij}=\frac{x_{ij}-b_{j}}{a_{j}-b_{j}}$$ (1)

Situation 2: for the cost attribute index (the smaller the index value, the better), the conversion formula is as follows:

$$y_{ij}=\frac{a_{j}-x_{ij}}{a_{j}-b_{j}}$$ (2)

Situation 3: for the interval type attribute index (index value falling into a certain range is the best condition), the conversion formula is as follows:

$$y_{ij}=\{\begin{array}{c} 1-\frac{q_{1}-x_{ij}}{\text{max}(q_{1}-b_{j},a_{j}-q_{2})},\;x_{ij}<q_{1}\\ 1-\frac{x_{ij}-q_{2}}{\text{max}(q_{1}-b_{j},a_{j}-q_{2})},\;x_{ij}>q_{2}\\ 1,\;q_{1}\leq x_{ij}\leq q_{2} \end{array}$$ (3)

Where [*q*_1_, *q*_2_] is the stable interval of the attribute index.

In the sections screening system of hazardous materials, benefit indexes are *c*_1_, *c*_3,_ *c*_4,_ *c*_7,_ and *c*_13_, the value of which is processed by dimensionless formula 1; cost indexes are *c*_2,_ *c*_5,_ *c*_6,_ *c*_9,_ *c*_10,_ *c*_11,_ *c*_12,_ and *c*_14,_ the value of which is processed by dimensionless formula 2; and interval indexes are *c*_8_ and *c*_*1*5_, the value of which is processed by dimensionless formula 3.

### Screening system optimization for hazardous materials transportation based on genetic algorithm

The screening system of the prohibited section for hazardous materials uses GA to optimize the initial weight and threshold value of the neural network. Thus, the optimized neural network model is better than the traditional neural network to perform road screening.

#### Population initialization

Each individual is a binary string that includes connection weights input and hidden layers, threshold of hidden layer, connection weights hidden and output layers, and threshold of output layer by using binary encoding. All weights and thresholds coding are connected as individual coding because each weight or threshold is coded by M-bit binary code.

#### Fitness function

When the neural network model is used to predict the section, the norm of the sample forecasting value and expected value error matrices are outputted as the objective function to make the residuals of the prediction value and expected value as small as possible. Fitness assignment function is used to sort fitness value.

#### Selection operator

Selection operation is used to simulate the biological phenomenon of selecting the normal optimization. The selection operator of the GA uses stochastic universal sampling (SUS). SUS provides extensions of zero bias and minimal individual. Set npointer as the number of selected individuals at the same interval distance. The distance of selecting the pointer is 1/npointer, and the position of the first pointer is determined by the uniform random number of [0, 1/npointer].

#### Crossover operator

Crossover operation simulates the reproductive phenomenon in the process of biological evolution through the intersection of two chromosomes to produce a new excellent variety. The crossover operator of the genetic algorithm adopts the single-point crossover operator.

#### Mutation operator

Mutation operation simulates genetic mutations caused by natural factors in the biological genetic environment. Mutation genes are produced by a certain probability, and the genes of mutation are selected by using random method. In the system with binary-encoded chromosome, a gene of a chromosome is changed randomly from 1 to 0, or from 0 to 1. The diversity of population in genetic types can be ensured by using mutation operation to search in the space as large as possible and avoid being trapped in a local solution. Thus, a high quality of the optimal solution can be achieved.

### Neural network training for screening system of hazardous materials transportation based on LM algorithm

LM algorithm is the combination of the gradient descent and Gauss–Newton methods, which use the approximate two-order derivative information. LM algorithm does not require excessive adjustment parameters, and its running speed is faster than the gradient descent method. When the LM algorithm is used to train the neural network, the weight adjustment formula is shown as follows:

$$\Delta W={\left(J^{T}J+\mu I\right)}^{-1}J^{T}E$$ (4)

where Δ*W* is the weight correction; E is the error; J is the Jacobian matrix of the error to weight differential; *μ* is a scalar identifying the learning method, which is Newton method or gradient method[31]. The research shows that the LM method can effectively solve the limitations of the traditional back-propagation neural network (BPNN) and shorten the training time.

### Road screening procedures for the prohibited section of hazardous materials based on GA-LM-NN

Step 1: determine the screening index system for prohibited section of hazardous materials.
Step 2: collect historical data and dimensionless processing of the input data.
Step 3: determine the structure of neural network.
Step 4: optimize the initial weights and thresholds of the neural network for prohibited section screening system by GA.
Step 5: train and test the weights and thresholds of the neural network by using the LM method. If the test is not qualified, return to step 3; otherwise, proceed to step 6.
Step 6: road screening of prohibited section in the transportation network based on trained GA-LM-NN model.
Step 7: identify the prohibited section set and alternative transportation sections set of hazardous materials.

**Fig 2:**
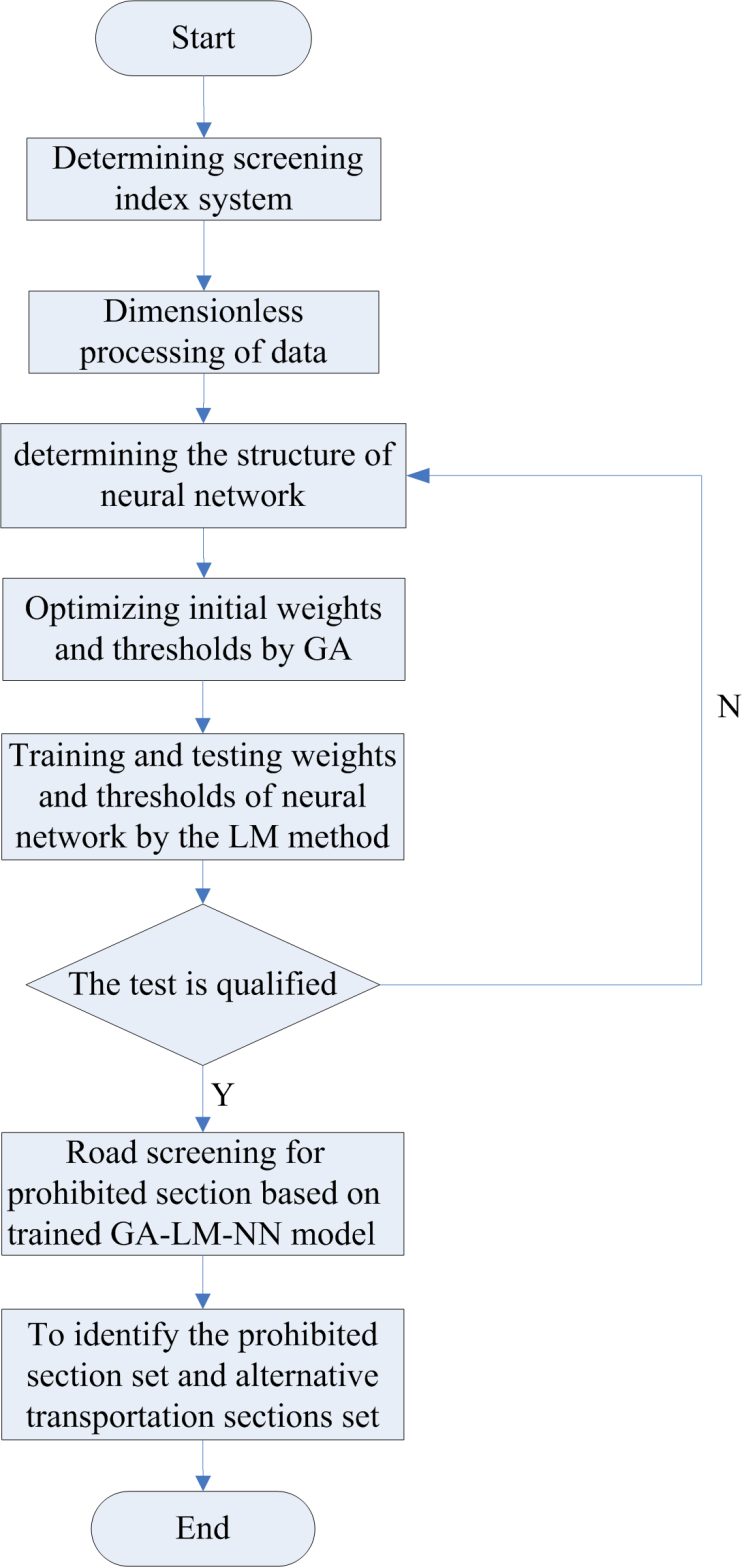
Screening process of prohibited section of hazardous materials based on GA-LM-NN.

The flow chart of the algorithm is shown in Fig 2.

Establishing the multi-objective optimization model and algorithm, and compiling the corresponding calculation program to determine the specific transportation route are necessary after obtaining the alternative transportation sections of hazardous materials.

## Multi-objective robust model of hazardous materials transportation vehicle routing

### Problem description

The distribution route optimization of hazardous materials implies the existence of a hazardous materials distribution center and multiple customers that require multiple vehicles distribution coordination to complete all distribution tasks. All vehicles are required to start from the distribution center. Each vehicle can serve multiple customers, and each customer needs one vehicle to be serviced. Each vehicle must return to the distribution center after completing the distribution task. Data information uncertainty in route optimization of hazardous materials transportation refers to the uncertainty of transportation time and transportation risk because decision-makers consider several influencing factors where the errors are caused by prediction methods and measurement tools.

The issue of hazardous materials transportation is more complex and requires higher safety requirements compared with general goods transportation. Thus, setting the minimum total risk in transportation of hazardous materials is necessary. Reducing transportation cost and resources consumption are essential in hazardous materials transportation. However, the length of transportation time is related directly to transportation cost. Long transportation time will increase the risk of hazardous materials transportation, thus setting a short transportation time is necessary. Therefore, for the hazardous materials vehicle routing problem, this paper can find several scientific routing scheme through the optimization of transportation risk and transport time to deliver hazardous materials safely and quickly to the customer demand point.

### Model building

#### Model assumption

Assuming that the supply of hazardous materials distribution centers is adequate, vehicle loading capacity is provided and the demand of each customer is specified, multiple vehicles of the distribution center can service the customer, the transportation risk and transportation time is identified among the customer demand points, and the customer demand point and distribution center is recognized but it is an uncertain number as interval number.

#### Symbol definition

The definition of the set is shown in Table 1.

**Table 1:** Set definition.

Set	Definition
*S*_*1*_	Set of customer demand point, where *S*_1_ = {*i|i* = 1, 2,…,*n*} shows that the number of customer demand points is *n* and the sequence number of nodes set is 1, 2,…,*n*
*S*	All nodes set in the transportation network, where *S* = *S*_*0*_ ⋃ *S*_*1*_
*V*	Available transportation vehicle set in the hazardous materials distribution center, where *V* = {*k|k* = 1, 2,…,*K*}
*E*	Road section set among nodes
*q*_*i*_	Demand of customer demand point *i*
$J_{i}^{r}$	Set of columns where all uncertain data ${\tilde{r}}_{ij}$ belonging to the *i*th row of the variable risk matrix, where $\left|J_{i}^{r}\right|\leq n$
$\psi_{i}^{r}$	Set of column subscript *j* of uncertain data ${\tilde{r}}_{ij}$ of line *i* in the variable risk matrix ${\tilde{r}}_{ij}$
$J_{i}^{t}$	Set of columns where all uncertain data ${\tilde{t}}_{ij}$ belonging to the *i*th row of the variable time matrix, where $\left|J_{i}^{t}\right|\leq n$
$\psi_{i}^{t}$	Set of column subscript *j* of uncertain data ${\tilde{r}}_{ij}$ of line *i* in variable time matrix ${\tilde{t}}_{ij}$

The definition of parameters is shown in Table 2.

**Table 2:** The parameter definition.

Parameter	Definition
*L*	Maximum load of transport vehicles
${\tilde{r}}_{ij}$	Variable transport risk from customer demand points *i* and *j*, where ${\tilde{r}}_{ij}\in [r_{ij},r_{ij}+{\hat{r}}_{ij}]({\hat{r}}_{ij}\geq 0)$
*r*_*ij*_	Transportation risk nominal value from customer demand points *i* and *j*
${\hat{r}}_{ij}$	Deviation of the variable transport risk to its nominal value from customer demand points *i* and *j*, where ${\hat{r}}_{ij}\geq 0$
*t*_*ij*_	Travel time nominal value from customer demand points *i* and *j*
${\hat{t}}_{ij}$	Deviation of variable travel time to its nominal value from customer demand points *i* and *j*, where ${\hat{t}}_{ij}\geq 0$
${\tilde{t}}_{ij}$	Variable transport risk from customer demand points *i* and *j*, where ${\tilde{t}}_{ij}\in [t_{ij},t_{ij}+{\hat{t}}_{ij}]({\hat{t}}_{ij}\geq 0)$
$\Gamma_{i}^{r}$	Parameter $\Gamma_{i}^{r}\in \left[0,\left|J_{i}^{r}\right|\right]$ to adjust robust risk of robust discrete optimization method and control the risk degree of conservatism, where decimal is permitted
$\left\lfloor \Gamma_{i}^{r}\right\rfloor$	Maximum integer less than $\Gamma_{i}^{r}$
$\Gamma_{i}^{t}$	Parameter $\Gamma_{i}^{t}\in \left[0,\left|J_{i}^{t}\right|\right]$ to adjust robust time of robust discrete optimization method and control the time degree of conservatism, where decimal is permitted
$\left\lfloor \Gamma_{i}^{t}\right\rfloor$	The maximum integer less than $\Gamma_{i}^{t}$

### Multi-objective robust optimization model of hazardous materials distribution route

$$\begin{array}{l} \min\;Z_{1}=\sum_{i\in S}\sum_{j\in S}\sum_{k\in V}r_{ij}x_{ijk}\\ +\underset{\left\{\Psi_{i}^{r}\cup \{m^{r}\}|\Psi_{i}^{r}\subseteq J_{i}^{r},\left|\Psi_{i}^{r}\right|=\left\lfloor \Gamma_{i}^{r}\right\rfloor ,m^{r}\in J_{i}^{r}\backslash \Psi_{i}^{r}\right\}}{\text{max}}\{\sum_{i\in S}\sum_{k\in V}\sum_{j\in \Psi_{i}^{r}}{\overset{⌢}{r}}_{ij}x_{ijk}+\sum_{i\in S}\sum_{k\in V}\sum_{m^{r}\in J_{i}^{r}\backslash \Psi_{i}^{r}}(\Gamma_{i}^{r}-\left\lfloor \Gamma_{i}^{r}\right\rfloor ){\overset{⌢}{r}}_{im^{r}}x_{im^{r}k}\}-\sum_{i\in S}^{}\sum_{k\in V}^{}r_{i0}x_{i0k} \end{array}$$ (5)

$$\begin{array}{l} \min\;Z_{2}=\sum_{i\in S}\sum_{j\in S}\sum_{k\in V}t_{ij}x_{ijk}\\ +\underset{\left\{\Psi_{i}^{t}\cup \{m^{t}\}|\Psi_{i}^{t}\subseteq J_{i},\left|\Psi_{i}^{t}\right|=\left\lfloor \Gamma_{i}^{t}\right\rfloor ,m^{t}\in J_{i}^{t}\backslash \Psi_{i}^{t}\right\}}{\text{max}}\{\sum_{i\in S}\sum_{k\in V}\sum_{j\in \Psi_{i}^{t}}{\overset{⌢}{t}}_{ij}x_{ijk}+\sum_{i\in S}\sum_{k\in V}\sum_{m^{t}\in J_{i}^{t}\backslash \Psi_{i}^{t}}(\Gamma_{i}^{t}-\left\lfloor \Gamma_{i}^{t}\right\rfloor ){\overset{⌢}{t}}_{im^{t}}x_{im^{t}k}\} \end{array}$$ (6)

s.t.

$$\sum_{i\in S}^{}g_{i}y_{ki}\leq L,\;\forall k\in V$$ (7)

$$\sum_{k\in V}^{}y_{ki}=1,\;\forall i\in S_{i}$$ (8)

$$\sum_{i\in S}^{}x_{ijk}=y_{kj},\;\forall j\in S,\;\forall k\in V$$ (9)

$$\sum_{j\in S}^{}x_{ijk}=y_{ki},\;\forall i\in S,\;\forall k\in V$$ (10)

$$X=\left(x_{ijk}\right)\in S$$ (11)

$$S=\{\left(x_{ijk}\right)|u_{i}-u_{j}+\text{n}x_{ijk}\leq n-1;1\leq i\neq j\leq n\}$$ (12)

$$\underset{(i,j)\in E}{\max}\{r_{ij}x_{ijk}\}\leq r,\;\forall k\in V$$ (13)

$$\underset{(i,j)\in E}{\max}\{\sum_{i\in S}^{}\sum_{j\in S}^{}r_{ij}x_{ijk}-\sum_{i\in S_{1}}^{}r_{i0}x_{i0k}\}\leq R,\;\forall k\in V$$ (14)

$$x_{ijk}=\left\{0,1\right\},\;\forall i\in S,\;\forall j\in S,\;\forall k\in V$$ (15)

$$y_{ki}=\{0,1\},\;\forall i\in S,\;\forall j\in S,\;\forall k\in V$$ (16)

where the objective function (5) expresses the minimization of hazardous materials transportation risks. The objective function (6) expresses the minimization of hazardous materials vehicle travel time. Constraint (7) expresses that the total tasks of vehicle *k* is not more than vehicle capacity. Constraint (8) expresses that task *i* is completed by one vehicle. Constraint (9) expresses the relationship of two variables. Constraints (11) and (12) are the branch elimination constraints, and $u_{i}=\{\begin{array}{cc} t & \;\text{Demand}\;\text{point}\;i\;\text{served}\;\text{by}\;\text{the}\;\text{hazardous}\;\text{materials}\;\text{transport}\;\text{vehicles}\;\text{with}\;t\;\text{step}\\ 0 & \text{otherwise} \end{array}$, $u_{j}=\{\begin{array}{cc} t & \;\text{Demand}\;\text{point}\;j\;\text{served}\;\text{by}\;\text{the}\;\text{hazardous}\;\text{materials}\;\text{transport}\;\text{vehicles}\;\text{with}\;t\;\text{step}\\ 0 & \text{otherwise} \end{array}$ Constraint (13) expresses that the transportation risk of each section must be less than or equal to threshold *r* set by decision makers. Constraint (14) expresses that the transportation risk of each route must be less than or equal to threshold *R* set by decision makers, whereas Constraints (15) and (16) express the decision variables constraint.

Each objective function of the above multi-objective robust model corresponds to parameter Γ. The purpose is to control the degree of conservatism of the solution. For example, $\Gamma_{i}^{r}$controls the risk conservative degree and reflects the decision maker’s risk preferences. When $\Gamma_{i}^{r}=0$, the max part objective function is equal to 0, and the model is the most sensitive to uncertain information, that is, when the weight of a road section changes in the transportation network, the optimal solution of the model is expected to change; with $\Gamma_{i}^{r}$ increasing gradually, sensitivity of the model to uncertain information is reduced and the obtained solution is robust[32].

In this part, the adjacency matrix of uncertain risk of transportation and time among the nodes is changed into one-dimensional matrix, specifically *m* = (*i*−1)*n*+*J*(1≤*i*≤*n*, 0≤*j*≤*n*), decision variables *x*_*ij*_ = *x*_*m*_, uncertain transportation risk ${\tilde{r}}_{ij}={\tilde{r}}_{m}$, uncertain transportation time ${\tilde{t}}_{ij}={\tilde{t}}_{m}$, and other corresponding basic data are changed in the form of subscript *m*. Certain uncertain parameters and set of parameters are changed through one-dimensional transformation. Theoretically, the corresponding transportation time also changes if the transportation risk between the two nodes changes. The robustness control parameters of transportation time and risk are controlled by using a control parameter for easier handling, that is, their values and change are consistent. Robustness control parameter Γ is in the interval [0, n^2^] after change because of 0≤*m*≤*n*^2^, which is defined by one integer. In general, the transportation risk and transport time nominal values between the two nodes decrease with *m* increasing and when *m* = (*i*−1)*n*+*i*(1≤*i*≤*n*), ${\hat{r}}_{m}=0$ and ${\hat{t}}_{m}=0$. Objective functions (5) and (6) of the robust model contain “max” extreme value problem, which are not beneficial for solving intuitively, applying the equivalent transformation of the expression containing max is necessary. Set feasible solution set *X*_*vrp*_ to satisfy all constraints, and robust discrete optimization criterion of the literature [33] will be used to change the multi-objective robust optimization model of hazardous materials distribution route in solving the following nominal problem.

Objective function is as follows:

$$\text{R}(r)=\hat{\Gamma }{\hat{r}}_{l}+\min(\sum_{m=1}^{n^{2}}r_{m}x_{{}_{m}}+\sum_{m=1}^{l}({\hat{r}}_{m}-{\hat{r}}_{l})x_{{}_{m}})$$ (17)

$$\text{T}(t)=\hat{\Gamma }{\hat{t}}_{l}+\min(\sum_{m=1}^{n^{2}}t_{m}x_{{}_{m}}+\sum_{m=1}^{l}({\hat{t}}_{m}-{\hat{t}}_{l})x_{{}_{m}})$$ (18)

Constraint condition is as follows:

$$x\in X_{vrp}$$ (19)

Then, the optimal objective function value can be obtained as ${\text{R}}^{*}=\underset{l=1,\cdots n^{2}+1}{\min}\text{R}(l)$ and ${\text{T}}^{*}=\underset{l=1,\cdots n^{2}+1}{\min}\text{T}(l)$.

## Improved multi-objective GA

Multiple objectives of multi-objective optimization may be in conflict with each other, which is different from single-objective optimization. The improvement of a sub-goal will lead to a decrease in another sub-target, that is, multiple sub-goals achieving optimum are impossible. Therefore, multi-objective optimization obtains a non-inferior solution set, the elements of which are called Pareto optimal or non-inferior optimal solutions. The Pareto optimal solution can also be interpreted as no solution exists better than at least one of the goals and not worse than other goals. The elements of the Pareto optimal solution set are not comparable to each other in terms of all objectives. Using the obtained Pareto set, decision makers selected one or many solutions from the Pareto optimal solutions as the optimal solution of multi-objective optimization problem according to other information or personal preference. Therefore, the main task of solving multi-objective optimization problem is to obtain widely distributed Pareto optimal solutions. In this paper, a multi-objective GA is designed to solve this model according to the multi-objective robust optimization model characteristics. The algorithm uses an improved selection strategy to complete the operation, applies partial matching cross transposition and single ortho swap methods to complete the operation of crossover and mutation, and employs the selected method to construct the Pareto optimal solution set.

**Fig 3:**
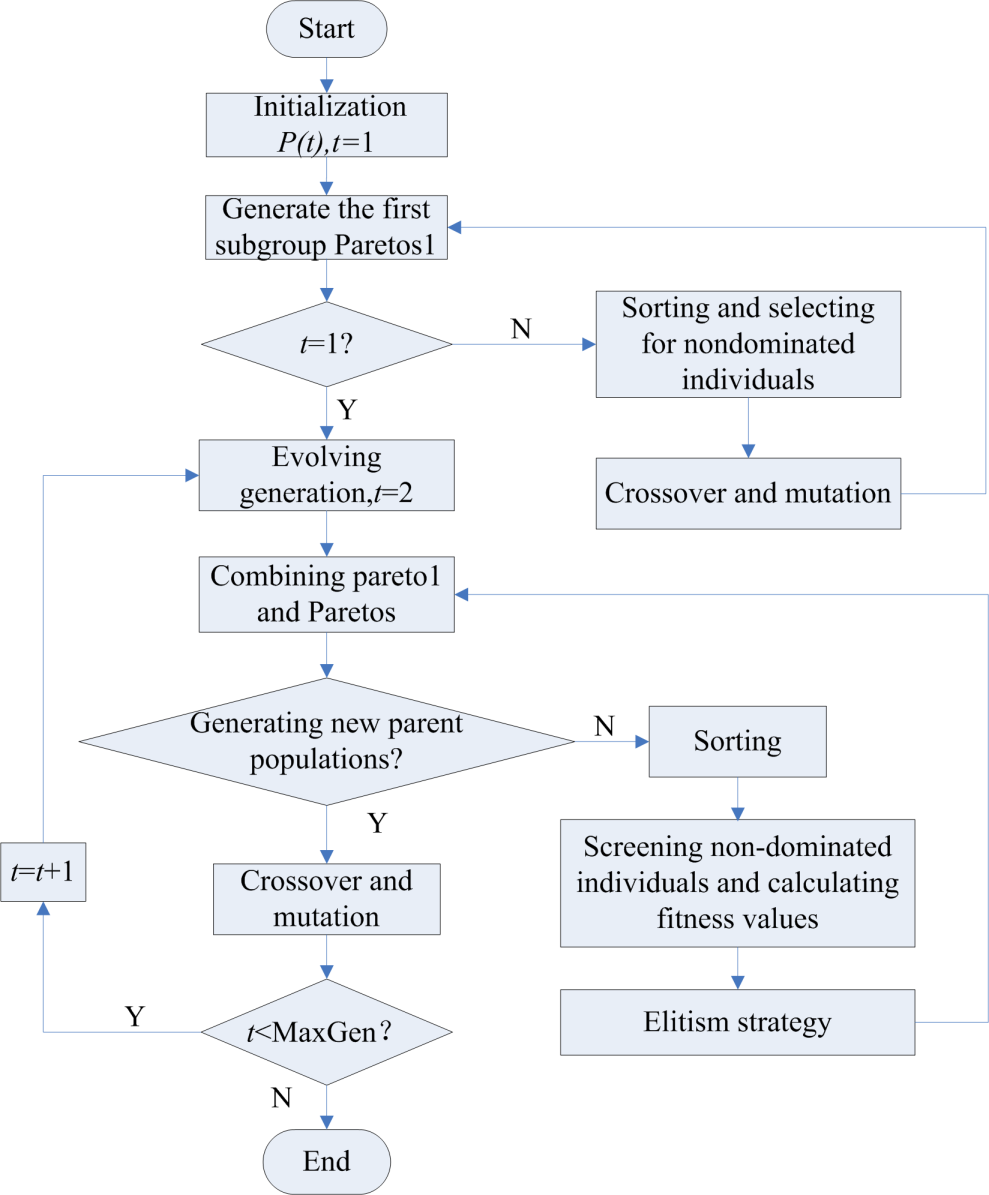
Flow diagram of the improved multi-objective GA.

The flow chart of the algorithm is shown in Fig 3.

### Chromosome encoding and decoding

In this section, natural number coding method is used. For example, the network containing one hazardous materials distribution center with number 0 and nine customers point, the initial population is generated by using the random generating method where the sequence 0 9 7 5 1 8 4 2 6 3 is one chromosome. This encoding method can ensure that each customer demand point is visited only one time. The model requires multiple vehicles to complete distribution services, and hence, the obtained chromosomes by this encoding must be decoded. Greedy strategy is used to decode the chromosome, and the specific method is as follows: customer demand points are inserted in the route according to the sequence of genes in the chromosome if it does not violate the load constraints. If load constraints are violated, another vehicle is required to service the customer. For example, the demands of nine customers: 2, 3, 3, 2, 2, 3, 4, 4, and 5 tons, and the maximum vehicle load is 8 tons, thus the decoding of chromosome is as follows:

Route of vehicle 1: 0→9→7→5→0;

Route of vehicle 2: 0→1→8→4→0;

Route of vehicle 3: 0→2→6→0;

Route of vehicle 4: 0→3→0.

### Improved elite selection operation

Step 1: Individual symbol domain, non-dominated set paretos, structure set paretos1, non-bad target set nds1, nds2 of population are initialized.
Step 2: Sorting population and different individuals of the population are replicated to construct set paretos1. The current non-dominated individuals obtained by exclusion method are inserted into non-dominated set paretos. The individual paretos and paretos0 (Pareto pool, storing all non dominated individuals) are placed in pareto pool, thereby ensuring that individuals of the Pareto pool are non-dominated individuals.
Step 3: Goal number *i* is generated randomly. Roulette selection is conducted based on objective function *i* for the entire population. Selected individuals are labeled in the corresponding marker domain flag[*i*], and the individuals copied to a non-dominated objective set ndsl. When comparing the individuals based on the elite retention rules, if an individual is the elite, the elite of the elite set aims is replaced as the individual.
Step 4: Goal number *j* is generated Randomly. Roulette selection is conducted based on objective function *j* for the entire population. Select individuals are labeled in the corresponding marker domain flag[*j*]. If a label is present in the marker domain for an individual, the individual will be copied to a non-dominated objective set ndsl. If two labels are present in the marker domain for an individual, the individual will be copied to the non-dominated objective set nds2. When comparing individuals under based on the elite retention rules, if an individual is the elite, the elite of elite set aims is replaced as the individual.
Step 5: The two individuals in the elite set aims to the next generation is copied.
Step 6: Non-inferior individuals are chosen. If *N*>|aims|+|paretod0|+|nds2|+|nds1|, all individuals of paretos0, nds2 and nds1 must be copied to the next generation, and generating randomly *N*−|aims|−|paretod0|−|nds2|−|nds1| individuals placed in the next generation, then exit is conducted. If *N* = |aims|+|paretod0|+|nds2|+|nds1|, all individuals of paretos0, nds2 and nds1 need to be copied to the next generation, then exit is conducted. If *N*>|aims|+|paretod0|+|nds2|, all individuals of paretos0 and nds2 are needed to be copied to next generation, and generating randomly *N*−|aims|−|paretod0|−|nds2|, individuals in nds1 placed in next generation, then exit is conducted. If *N* = |aims|+|paretod0|+|nds2|, all individuals of paretos0 and nds2 are needed to be copied to the next generation, then exit is conducted. If *N*>|aims|+|paretod0|, all individuals of paretos0 must be copied to next generation, and generating randomly *N*−|aims|−|paretod0| individuals in nds2 placed in the next generation, then exit is conducted. If *N* = |aims|+|paretod0|, all individuals of paretos0 are needed to be copied to the next generation, then exit is conducted. Otherwise, generating randomly *N*−|aims| individuals in paretos0 placed in the next generation. In the process, *N*, |aims|, |paretod0|, |nds1| and |nds2| express the size of the population, the size of elite set, the number of non-dominated individuals in the pareto pool, the individual number of non-inferior target set nds1 and the individual number of non-inferior target set nds2, respectively.

### Crossover operation

The partial match crossover shift method is used to complete the crossover operation. Specific steps as follows:

Step 1: *A* mating area is chosen randomly in two selected chromosomes, such as *A* = 0 9 7 I 5 1 8 4 I 6 3, *B* = 0 6 5 I 9 2 1 7 8 I 4 3.
Step 2: Mating area of chromosome *B* is inserted into chromosome *A*, and mating area of chromosome *A* is inserted into chromosome *B*. For example, the two chromosomes in step 1 are changed into such as *A'* = 0 9 2 1 7 8 I 9 7 5 1 8 4 2 6 3, *B'* = 0 5 1 8 4 2 I 6 5 9 2 1 7 8 4 3 after step 2.
Step 3: Customer demand points, which are the same as that of mating area in the self-mating region of chromosome *A'* and *B'*, are deleted and chromosomes obtained are *A"* = 0 9 2 1 7 8 5 4 6 3, *B"* = 0 5 1 8 4 2 6 9 7 3.

### Mutation operation

The single ortho swap method is used to complete the mutation operation: two different gene positions are selected randomly in parental chromosomes, and the position of the swap starting and ending are determined based on the sequence of two genes. If the number of gene between the starting and ending positions is even, all odd numbered genes in this range and its right genetic exchange are used. If the number is odd, the last odd numbered gene is not changed, and the remaining odd numbered gene and its right genetic exchange are used.

### Constructing pareto optimal set

The exclusive method is used to construct the non-dominated set as follows:

Step 1: The non-dominated set paretos and constructive set paretos1 are initialized.
Step 2: All different individuals of population pops are copied to paretos1 in order.
Step 3: The different individuals of constructive set paretos1 *X* are compared with other individuals *Y* after them. If *X* dominates *Y*, then *Y* is removed from constructive set paretos1; if *Y* dominates *X*, *X* is removed, then exit is conducted, and the next comparison is begun. After comparison, *X* is non-dominated and *X* is copied to non-dominated set paretos if *X* is not dominated by any other individual.
Step 4: The value of *X* is assigned by that of the individual behind it in constructive set paretos1.
Step 5: Steps 3 and 4 are repeated, until individual *X* is the last one of constructive set paretos1.
Step 6: The last individual of paretos1 is copied to non-dominated set in paretos.

The individuals of the non-dominated set constructed by the above method, in any case, are non-dominated. However, this is only the non-dominated set of the current generation. We know that non-dominated individuals of the current generation are not global; thus, each individual of the current non-dominated set paretos should be compared with all individuals of paretos0 (pareto pool, which stores all non-dominated individuals until the present) to judge whether it is the global non-dominated individual, and then we implement the corresponding operation.

## Case study

We study the Zhengzhou coal materials supply and marketing company, which is responsible for distributing explosives for the 15 coal mines of Zhengzhou Coal Group in China, such as Dragon, Cui Miao, Lu Gou, and so on. The company uses joint distribution method in which a vehicle can service multiple spots. A total of 32 roads are in the distribution area, and these roads must be selected to complete optimal distribution route choice. The maximum load of each vehicle is 8 tons, and supply is adequate. A total of 15 demand points are used, which are shown in Table 3.

**Table 3:** Demand points of all customers.

Customer demand points	1	2	3	4	5	6	7	8	9	10	11	12	13	14	15
Demand (ton)	2.5	1	4	2	2	3.5	2	3.5	2.5	1	4	3.5	1	3	2.5

Data on the 32 roads are collected and processed via dimensionless processing, the results of which are shown in Table 4. The values can be used as input data of neural network. In addition to collecting data in the table, collecting the historical data of traffic accidents and traffic safety from the local traffic police, traffic bureau, and other departments is also necessary. Serious or severe traffic accidents occurred in roads *R*24, *R*25, *R*27, *R*29, and *R*32 recently, and hence these roads are considered as unsafe roads. General traffic accidents occurred in roads *R*22, *R*23, *R*26, and *R*31 recently, thus, there roads are generally safe roads. General traffic accidents occurred in in roads *R*20, *R*21, *R*28, and *R*30 recently, and hence, these roads are viewed as safe roads.

**Table 4:** Road attribute data.

Road	*c*_*1*_	*c*_*2*_	*c*_*3*_	*c*_*4*_	*c*_*5*_	*c*_*6*_	*c*_*7*_	*c*_*8*_	*c*_*9*_	*c*_*10*_	*c*_*11*_	*c*_*12*_	*c*_*13*_	*c*_*14*_	*c*_*15*_
*R*1	0.015	0.75	0.080	0.207	0.75	0.8	0.08	1	1	1	1	1	1	1	1
*R*2	0	0.5	0.195	0.138	0.85	1	0.2	1	0.5	0.5	0.75	0.75	0.88	0.5	1
*R*3	0.508	0.25	0.310	0.138	0.5	0.5	0	1	0.5	0.5	0.5	0.5	0.88	0.5	1
*R*4	0.754	0.25	0.540	0.483	0.65	0.7	0.6	1	1	1	0.75	1	1	1	1
*R*5	0	0	0	0	0	0	0	0.5	0	0	0	0.25	0	0	0.5
*R*6	1	1	0.885	1	1	1	1	0	1	1	0.75	1	1	1	0
*R*7	0.569	0.75	0.540	0.310	0.75	0.5	0.08	1	0.5	0.5	0.75	0.75	0.8	0	1
*R*8	0.631	0.25	0.310	0.241	0.5	0.8	0.6	1	0.5	0.5	0.25	0	1	0	0.5
*R*9	0.015	0.75	0.057	0.034	0.5	0.4	0.08	0.5	1	1	0.75	0.75	0	1	0.5
*R*10	0.754	0	1	1.172	0.5	0.8	1	0	0.5	0.5	0.25	0.25	1	1	0
*R*11	0.031	1	0.195	0.552	0.25	1	0.4	1	1	1	0.75	0.75	1	1	1
*R*12	0.266	0.5	0.425	0.483	0	0.6	0.08	1	1	0.5	0.25	0.5	0.8	0.5	0.75
*R*13	1	1	0.885	1	1	1	1	0	1	1	0.25	0	0	0	0.25
*R*14	0.028	0.25	0.195	0.069	0.85	1	0	1	0.5	1	0	0.25	0	0	0.5
*R*15	0	0	0.137	0.241	0.5	0.5	0	1	0	0.5	0.25	0.25	0	0	0.5
*R*16	0.031	0	0.080	0.138	0.05	0.7	0	1	0.5	1	0.5	0.5	0	0.5	0
*R*17	0.262	0.75	0.310	0.655	0.6	0.9	0.08	1	1	0.5	0.75	0.75	0.88	0.5	1
*R*18	0.508	0.75	0.540	0.966	0.25	0.9	0.6	0	1	0.5	1	1	1	1	0.5
*R*19	0.262	0.5	0.252	0.931	0.9	0.9	0.6	0	1	1	1	1	0	1	0.5
*R*20	0.508	0.75	0.885	0.655	0	1	0.8	0	1	1	0.75	1	1	1	1
*R*21	1	1	1	1	0.95	1	1	0	0.5	1	1	1	1	1	1
*R*22	0.508	0.75	0.942	0.931	0.75	0.9	0.8	1	0.5	0.5	0.5	1	1	0	1
*R*23	0.508	1	0.977	0.897	0.65	0.9	0.8	1	0	0.5	0.75	1	1	1	0.5
*R*24	0.262	0.5	0.461	0.621	0.2	0.8	1	1	0.5	1	0.75	0.75	0.88	0.5	0
*R*25	0.262	0.5	0.483	0.690	0.1	1	1	1	1	0.5	0.75	0.75	0.8	0.5	1
*R*26	0.231	0.25	0.333	0.759	0.115	1	0.08	1	1	0.5	0.5	0.75	0	0.5	0.5
*R*27	0.231	0.5	0.207	0.931	0.905	1	0	1	0	0.5	0.5	0.75	0	1	1
*R*28	0.538	0.75	0.885	0.724	0.886	0.9	0.8	1	1	1	1	1	1	0.5	1
*R*29	0.538	0.75	0.954	0.621	0.1	1	0.8	1	1	1	1	1	1	1	1
*R*30	1	1	1	0.999	0	1	1	0	1	0.5	1	1	1	1	1
*R*31	0.508	0.75	0.425	1	0.1	0.9	0.6	1	0.5	1	1	0.75	1	1	1
*R*32	0	0.25	0.092	0.038	0	1	0.08	0.5	0	0.5	0.25	0.25	0	0.5	1

Transportation route of hazardous materials are selected based on the constructed GA-LM-NN screening algorithm. The transfer function of neural network hidden layer neuron uses *S* tangent function tansig () and transfer function of output layer neurons uses *S* logarithmic function logsig () because the output mode is 0–1, to meet the requirements of network output expressly. The optimizing the screening system is attained via genetic algorithm. The parameters of the genetic algorithm are set as shown in Table 5.

**Table 5:** Operating parameters of genetic algorithm.

Parameter name	Population size	Maximum genetic generations	Binary digit of variable	Crossover probability	Mutation probability
Value	40	80	10	0.7	0.01

**Fig 4:**
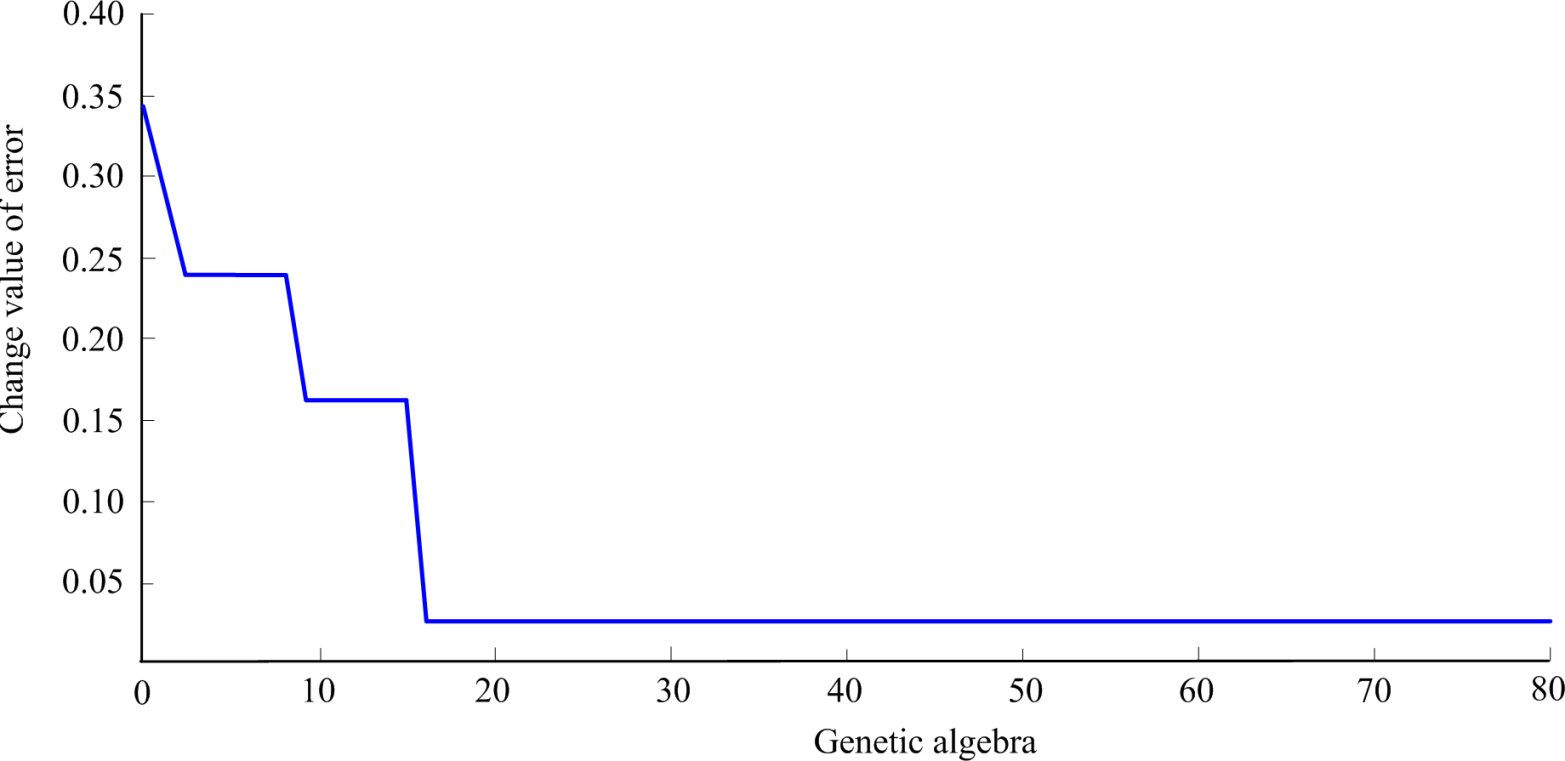
Error evolution curve.

Optimized weights and thresholds are obtained after genetic algorithm. The minimum error is err = 0.074296. The evolution curve obtained is shown in Fig 4.

Optimized screening system neural network model is trained and tested. The R20–R29 data are used to train the neural network, whereas R30–R32 data are used to test the accuracy of neural network model. Simple calculation shows that 558 weights and 34 thresholds are present in the neural network. Therefore, the number of genetic algorithm optimization parameters is 592. The norm of samples test error is regarded as a measure of the generalization ability of the network (network quality), and the individual fitness value is calculated via error norm. The smaller the error norm, the greater the individual fitness value, and the more excellent individuals are present.

After the optimal initial weights and threshold are obtained via GA; the initial weights and thresholds are introduced into the network to draw the training error curve, forecasting value, forecast error, and training error. The simulation program is compiled, and the results of the GA-LM-NN model simulation are compared with the results of BPNN model.

By comparison, the test sample error after optimization of initial weights and thresholds is reduced from 1.251 to 0.074296, and the error of training samples is reduced from 0.36186 to 0.091869. Therefore, the road screening GA-LM-NN model, which is optimized by GA, has caused significant improvement in accuracy, compared with the pure BPNN model.

Transportation road screening is simulated based on the GA-LM-NN model, and the transportation road set obtained is {R1, R2, R3, R4, R7, R9, R11, R12, R17, R19, R20, R21, R22, R23, R26. R28, R30, R31}. Transportation risk and transportation time between points are calculated based on the set, as shown in Tables 6–7. The two tables show the risk nominal and time nominal values, respectively. Some deviations occur in obtaining the nominal value because of various reasons. Set transportation risk deviation is ${\hat{r}}_{m}(0\leq {\hat{r}}_{m}<0.5r_{m})$, and transportation time deviation is ${\hat{t}}_{m}(0\leq {\hat{t}}_{m}<0.5r_{m})$. The nominal value and deviation consist of the basic data of transportation route multi-objective robust model of single distribution center.

**Table 6:** Nominal transport risk value of hazardous materials.

*r*_*ij*_	0	1	2	3	4	5	6	7	8	9	10	11	12	13	14	15
0	0	40	80	53	97	81	74	64	103	98	93	52	100	90	76	61
1	40	0	41	46	65	50	51	110	71	48	40	40	66	77	80	82
2	80	41	0	83	51	87	72	65	44	83	95	96	76	61	102	91
3	53	46	83	0	107	105	78	50	72	56	101	54	95	99	110	110
4	97	65	51	107	0	83	67	58	61	99	41	66	46	88	43	40
5	81	50	87	105	83	0	105	59	59	81	89	99	91	74	54	92
6	74	51	72	78	67	105	0	73	72	107	92	47	82	67	92	83
7	64	110	65	50	58	59	73	0	80	65	50	55	70	97	76	110
8	103	71	44	72	61	59	72	80	0	93	64	51	86	74	44	89
9	98	48	83	56	99	81	107	65	93	0	75	50	107	50	104	89
10	93	40	95	101	41	89	92	50	64	75	0	61	70	44	108	88
11	52	40	96	54	66	99	47	55	51	50	61	0	50	102	98	81
12	100	66	76	95	46	91	82	70	86	107	70	50	0	53	52	98
13	90	77	61	99	88	74	67	97	74	50	44	102	53	0	73	51
14	76	80	102	110	43	54	92	76	44	104	108	98	52	73	0	75
15	61	82	91	110	40	92	83	110	89	89	88	81	98	51	75	0

**Table 7:** Nominal transport time value of hazardous materials.

*t*_*ij*_	0	1	2	3	4	5	6	7	8	9	10	11	12	13	14	15
0	0	143	97	79	120	136	146	141	107	57	91	157	44	112	133	100
1	143	0	54	173	169	117	88	106	92	159	84	104	78	178	81	144
2	97	54	0	119	67	147	158	96	110	165	43	180	120	47	114	67
3	79	173	119	0	158	128	132	67	158	57	55	144	84	172	80	87
4	120	169	67	158	0	59	143	157	139	124	145	75	60	40	48	153
5	136	117	147	128	59	0	160	69	56	118	42	56	104	146	136	116
6	146	88	158	132	143	160	0	50	101	68	138	80	101	72	121	115
7	141	106	96	67	157	69	50	0	128	62	111	175	138	170	66	87
8	107	92	110	158	139	56	101	128	0	65	180	104	180	53	128	53
9	57	159	165	57	124	118	68	62	65	0	101	171	46	166	80	72
10	91	84	43	55	145	42	138	111	180	101	0	148	97	68	128	125
11	157	104	180	144	75	56	80	175	104	171	148	0	103	105	124	129
12	44	78	120	84	60	104	101	138	180	46	97	103	0	160	156	128
13	112	178	47	172	40	146	72	170	53	166	68	105	160	0	141	119
14	133	81	114	80	48	136	121	66	128	80	128	124	156	141	0	92
15	100	144	67	87	153	116	115	87	53	72	125	129	128	119	92	0

**Fig 5:**
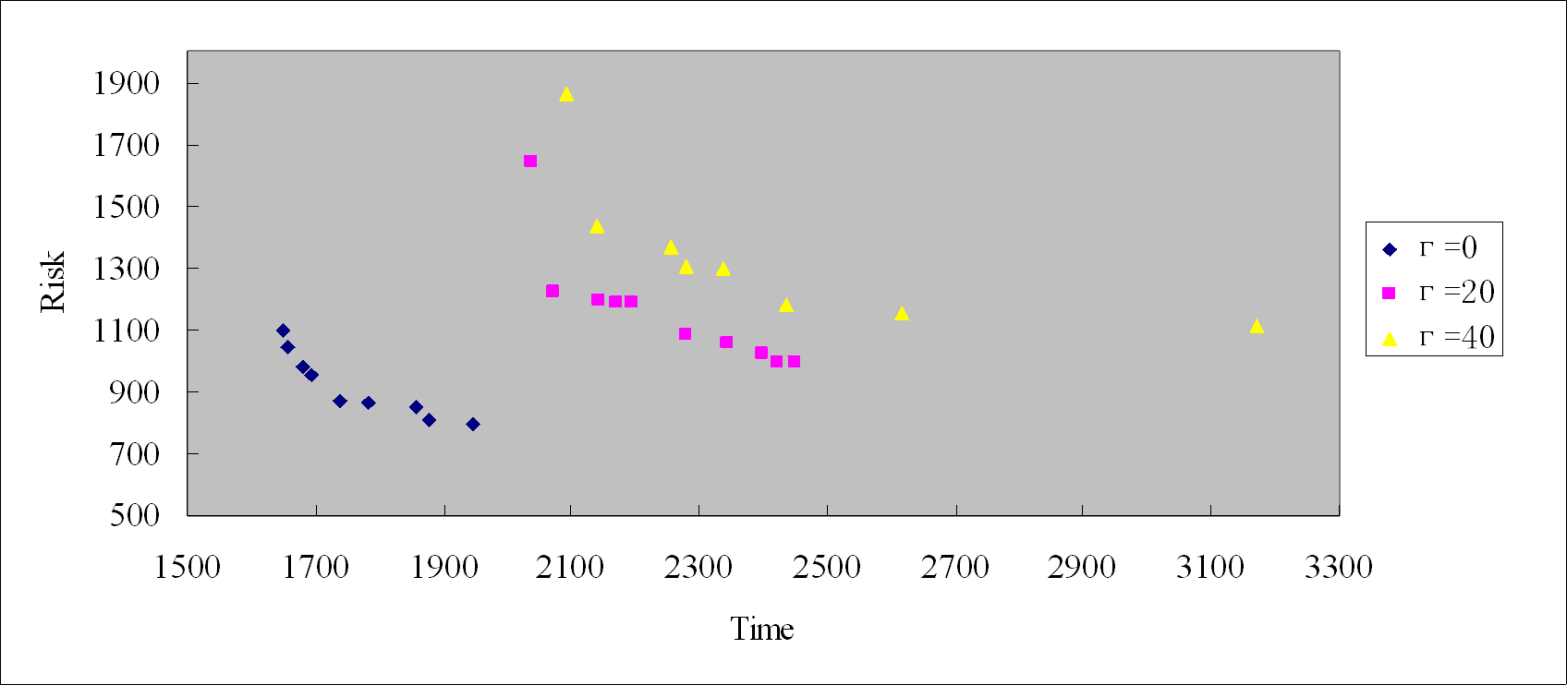
Pareto optimal solution distribution of robust control parameters Γ = 0, Γ 20 and Γ = 40.

We set the size of population PopSize = 200, Evolutionary generation MaxGen = 200, Crossover rate CrossRate = 0.95, and Mutation rate MutationRate = 0.1. The Pareto solution set with different robust control parameters can be obtained by calculation, which are shower in Tables 8–11 and Fig 5.

**Table 8:** Pareto solution set of robust control parameters Γ = 0.

Chromosome decoding route	Transportation risk	Transportation time
0 6 7 9 0 15 14 4 0 3 10 5 0 11 12 0 1 2 13 8	952	1695
0 13 8 5 10 0 7 6 9 0 11 1 2 0 12 3 0 4 14 15	1099	1649
0 14 4 5 10 0 12 1 2 13 0 11 8 0 6 7 9 0 15 3	1045	1657
0 11 12 0 14 4 5 10 0 1 2 13 8 0 7 6 15 0 9 3	983	1680
0 15 4 12 0 14 7 5 10 0 1 2 13 6 0 11 8 0 3 9	868	1781
0 14 5 7 10 0 11 6 0 1 2 13 8 0 15 4 12 0 3 9	810	1877
0 15 4 12 0 14 5 7 10 0 11 6 0 1 2 8 13 0 3 9	793	1945
0 14 7 5 10 0 1 2 13 8 0 3 9 0 15 4 12 0 11 6	871	1738
0 3 9 0 15 4 12 0 7 14 5 10 0 1 2 13 6 0 11 8	851	1856

**Table 9:** Pareto solution set of robust control parameters Γ = 20.

Chromosome decoding route	Transportation risk	Transportation time
0 1 2 13 10 9 0 15 14 4 0 6 8 0 11 12 0 3 7 5	1021	2398
0 1 2 13 10 9 0 8 6 0 11 12 0 15 14 4 0 3 7 5	1084	2280
0 1 2 13 10 9 0 6 8 0 11 12 0 14 4 15 0 3 7 5	993	2423
0 10 8 9 0 15 4 14 0 2 1 6 13 0 11 12 0 3 7 5	1188	2172
0 10 8 9 0 15 14 4 0 1 2 13 6 0 11 12 0 3 7 5	1196	2144
0 1 2 13 10 9 0 4 14 15 0 6 8 0 11 12 0 3 7 5	1057	2345
0 12 1 2 10 0 6 11 0 13 8 9 0 4 14 15 0 5 7 3	1643	2038
0 10 2 1 12 0 6 9 13 0 15 14 4 0 11 8 0 3 7 5	1187	2196
0 13 8 9 0 12 10 1 2 0 15 4 14 0 6 11 0 3 7 5	1226	2073
0 1 2 13 10 9 0 14 4 15 0 6 8 0 11 12 0 3 7 5	993	2451

**Table 10:** Pareto solution set of robust control parameters Γ = 40.

Chromosome decoding route	Transportation risk	Transportation time
0 13 2 1 5 10 0 11 3 0 14 4 9 0 7 15 8 0 6 12	1367	2254
0 9 12 4 0 11 6 0 3 7 5 0 15 13 14 0 8 2 1 10	1155	2615
0 13 2 1 5 10 0 11 3 0 9 7 6 0 15 8 4 0 12 14	1307	2278
0 6 13 2 4 0 14 7 10 0 15 9 0 11 12 0 1 3 0 5 8	1183	2436
0 2 1 13 8 0 6 11 0 14 4 5 10 0 15 3 0 7 9 12	1860	2093
0 14 5 7 0 11 12 0 9 13 2 6 0 3 1 10 0 15 4 8	1115	3169
0 5 8 9 0 3 11 0 7 4 12 0 15 14 13 10 0 2 1 6	1295	2337
0 13 2 4 1 10 0 5 8 15 0 6 11 0 14 3 0 7 9 12	1433	2140

**Table 11:** Objective optimal solutions of robust control parameters Γ = 0, Γ = 20 and Γ = 40.

Optimal route	Γ = 0	Γ = 20	Γ = 40
Optimal risk route	0 15 4 12 0	0 1 2 13 10 9 0	0 14 5 7 0
	0 14 5 7 10 0	0 6 8 0	0 11 12 0
	0 11 6 0	0 11 12 0	0 9 13 2 6 0
	0 1 2 8 13 0	0 14 4 15 0	0 3 1 10 0
	0 3 9 0	0 3 7 5 0	0 15 4 8 0
Optimal time route	0 13 8 5 10 0	0 12 1 2 10 0	0 2 1 13 8 0
	0 7 6 9 0	0 6 11 0	0 6 11 0
	0 11 1 2 0	0 13 8 9 0	0 14 4 5 10 0
	0 12 3 0	0 4 14 15 0	0 15 3 0
	0 4 14 15 0	0 5 7 3 0	0 7 9 12 0

Tables 8–10 show the optimal solution set with robustness control parameters Γ = 0, Γ 20 and Γ = 40, respectively. Ordered string of chromosome decoding route refers to the chromosome decoding sequence based on greedy strategy of multi-objective genetic algorithm, in which each 0 shows a vehicle, and natural numbers behind 0 show the customer demand point and order. For example, the first decoding sequence of Table 8 means it needs 5 vehicles, and each vehicle corresponds to a sub route, respectively: 0→6→7→9→0, 0→15→14→4→0, 0→3→10→5→0, 0→3→10→5→0, 0→11→12→0, 00→1→2→13→8→0. All transportation vehicles start from the distribution center 0, through demand points, and finally return to distribution center 0. Pareto solutions are not comparable with each other from Tables 8–10. Determining the optimal solution can be difficult, and decision makers need other conditions to select suitable routes in Pareto solution sets with high robustness. Fig 5 shows optimal Pareto solution set distribution with robust control parameters Γ = 0, Γ 20 and Γ = 40, thereby illustrating that Pareto solutions are found by multi-objective genetic algorithm. Table 11 shows Pareto extreme solutions (two endpoints of Pareto curve) of Pareto solution with robust control parameters Γ = 0, Γ 20 and Γ = 40, namely, the corresponding transportation vehicle route of Tables 8–10 when each single objective is optimal, respectively. Table 11 shows that when the robust control parameter Γ increases, the Pareto solutions become more robust.

The strength Pareto genetic algorithm (SPEA) is used to test the efficiency of the improved multi-objective genetic algorithm. The algorithm parameter and Pareto optimal solution set selection strategy are the same as those of the improved multi-objective genetic algorithm designed in this paper. The results are shown in Table 12. Compared with SPEA and under different values, the mean values of two objective functions obtained from the improved multi-objective genetic algorithm designed in this paper are better, and the operation time is reduced. The results show that the improved multi-objective genetic algorithm designed in this paper can not only obtain a more satisfactory solution, but also has faster convergence speed compared with the traditional genetic algorithm.

**Table 12:** Performance comparison between improved multi-objective genetic algorithm and SPEA.

Optimization objective	Improved multi-objective genetic algorithm			SPEA		
Γ	0	20	40	0	20	40
Mean value of risk objective	919	1159	1339	1018	1271	1428
Mean value of time objective	1764	2252	2415	1895	2366	2571
Run time/(s)	4	6	8	5	7	11

## Conclusion

Optimization of distribution route is an important link to ensure safe transportation of hazardous materials. Scientific and reasonable distribution route design of hazardous materials can make hazardous materials reach the customer demand point safely, quickly, and economically. However, the optimized scheme may incur serious security risks if road screening is not carried out before route optimization. This paper extensively studies the problem of road screening for hazardous materials transportation, and builds road screening algorithm based on GA-LM-NN and the multi-objective robust optimization model of transportation route with adjustable robustness based on Bertsimas. The improved elitist selection strategy is used to complete choice operation, partial matching cross shift method, and single ortho swap method is used to complete crossover and mutation operation. The Pareto optimal solution set is constructed based on the exclusive method. The study shows that the proposed GA-LM-NN road screening algorithm can determine quickly the suitable transportation section sets of hazardous materials. Furthermore, transportation path multi-objective robust optimization model and algorithm can determine rapidly the Pareto solution set of different robustness transportation route. Finally, decision makers can choose suitable transportation routes from better robust Pareto solutions based on actual situation or preferences through a case study.

The establishment of the visual road screening for hazardous materials transportation and route robust optimization platforms based on geographic information system will be the focus of future research.
